# Deletion of the Small RNA Chaperone Protein Hfq down Regulates Genes Related to Virulence and Confers Protection against Wild-Type *Brucella* Challenge in Mice

**DOI:** 10.3389/fmicb.2015.01570

**Published:** 2016-01-20

**Authors:** Shuangshuang Lei, Zhijun Zhong, Yuehua Ke, Mingjuan Yang, Xiaoyang Xu, Hang Ren, Chang An, Jiuyun Yuan, Jiuxuan Yu, Jie Xu, Yefeng Qiu, Yanchun Shi, Yufei Wang, Guangneng Peng, Zeliang Chen

**Affiliations:** ^1^Key Laboratory of Animal Disease and Human Health of Sichuan Province, College of Veterinary Medicine, Sichuan Agricultural UniversityChengdu, China; ^2^Institute of Disease Control and Prevention, Academy of Military Medical ScienceBeijing, China; ^3^Inner Mongolia Key Laboratory of Molecular Biology, Inner Mongolia Medical UniversityHohhot, China; ^4^Experimental Animal Center, Academy of Medical SciencesBeijing, China; ^5^Department of Laboratory Medicine, The General Hospital of Chinese People's Armed Police ForcesBeijing, China; ^6^College of Medicine, Shihezi UniversityShihezi, China

**Keywords:** brucellosis, *hfq*, virulence-related genes, host immune response, protective immunity

## Abstract

Brucellosis is one of the most common zoonotic epidemics worldwide. *Brucella*, the etiological pathogen of brucellosis, has unique virulence characteristics, including the ability to survive within the host cell. Hfq is a bacterial chaperone protein that is involved in the survival of the pathogen under stress conditions. Moreover, *hfq* affects the expression of a large number of target genes. In the present study, we characterized the expression and regulatory patterns of the target genes of Hfq during brucellosis. The results revealed that *hfq* expression is highly induced in macrophages at the early infection stage and at the late stage of mouse infection. Several genes related to virulence, including *omp25, omp31, vjbR, htrA, gntR*, and *dnaK*, were found to be regulated by *hfq* during infection in BALB/c mice. Gene expression and cytokine secretion analysis revealed that an *hfq*-deletion mutant induced different cytokine profiles compared with that induced by 16M. Infection with the *hfq*-deletion mutant induced protective immune responses against 16M challenge. Together, these results suggest that *hfq* is induced during infection and its deletion results in significant attenuation which affects the host immune response caused by *Brucella* infection. By regulating genes related to virulence, *hfq* promotes the virulence of *Brucella*. The unique characteristics of the *hfq*-deletion mutant, including its decreased virulence and the ability to induce protective immune response upon infection, suggest that it represents an attractive candidate for the design of a live attenuated vaccine against *Brucella*.

## Introduction

Brucellosis is a common zoonotic epidemic worldwide, especially in developing countries. Over the years, brucellosis has caused great loss to agriculture and human health. In recent years, the incidence of brucellosis has increased steadily in many countries (Pappas et al., 2006). Therefore, brucellosis has been defined as a reemerging infectious disease (Godfroid et al., 2005). Moreover, brucellosis has evolved from being an endemic occupational disease to a travel-associated zoonosis (Memish and Balkhy, 2004; Seleem et al., 2010). *Brucella*, the causative organism of brucellosis, has unique virulence characteristics; it has no plasmids and toxins, and intracellular survival is one of its most important virulence mechanisms (Atluri et al., 2011). To survive in host cells, *Brucella* needs to inhibit the host immune response, which is detrimental to bacterial survival and replication. To protect itself from the host immune system, *Brucella* limits its exposure to the innate and adaptive immune responses. These unique characteristics of *Brucella* have limited the development of effective vaccines and therapeutics for brucellosis.

Several proteins are involved in mediating the virulence of *Brucella*. It is possible that the proteins involved in virulence also affect the immune induction characteristics of the strain. Hfq is a bacterial chaperone protein that mediates RNA-RNA interactions and regulates gene expression at the post-transcriptional level (Valentin-Hansen et al., 2004). Hfq was first discovered in nonpathogenic *Escherichia coli*, and subsequently, in many pathogenic bacteria (Chao and Vogel, 2010). Hfq functions as a modulator of gene expression and participates in a variety of physiological processes, including modulating RNA transcription, folding, translation, and turnover (Waters and Storz, 2009). In most cases, *hfq* mediates sRNA-mRNA interactions to regulate gene expression, either positively or negatively (Vogel and Luisi, 2011). The pleiotrophic phenotype changes that result from the deletion of *hfq* have demonstrated that *hfq* is involved in stress resistance and infection.

*Brucella* has been shown to produce the Hfq protein (Bøggild et al., 2009; Cui et al., 2013). Hfq has been identified and characterized in several *Brucella* species (Bøggild et al., 2009; Caswell et al., 2012; Cui et al., 2013; Zhang et al., 2013). Deletion of *hfq* resulted in reduced survival of the mutant under conditions of stress, indicating that *hfq* is involved in adaptation to intracellular environments (Roop et al., 2003). Infection assays revealed that the deletion mutant had reduced survival capability in macrophages and mice. Hfq has been shown to coordinate expression of the virB type IV secretion system and BabR in *B. abortus* (Caswell et al., 2012). Transcription and proteomic analyses revealed that *hfq* affects the expression of a large number of target genes (Roop et al., 2003; Saadeh et al., 2015). Most recently, Hfq associated RNAs have been identified, providing more details about its regulation mechanism (Saadeh et al., 2015).

Several studies have demonstrated that the deletion of a gene related to virulence decreases the survival capability of *Brucella* (Edmonds et al., 2002; Haine et al., 2005; Caro-Hernández et al., 2007; Uzureau et al., 2007; Zhang et al., 2013). The decreased survival of the mutant strain alters the interaction between *Brucella* and the host (Salcedo et al., 2013). Immunization of the *hfq* mutant confers protection against *B. melitensis* challenge (Edmonds et al., 2002). Investigation of the mechanisms underlying this interaction will provide functional information on *hfq* and further evaluation of the vaccine candidate. Therefore, in the present study, we analyzed the expression profiles of *hfq* under both *in vitro* and *in vivo* conditions. In addition, some genes that are known to function in virulence, including *omp25* (Edmonds et al., 2002), *omp31* (Caro-Hernández et al., 2007), *vjbR* (Uzureau et al., 2007), *htrA* (Elzer et al., 1996), *gntR* (Haine et al., 2005), and *dnaK* (Köhler et al., 1996), were analyzed for their expression profiles in a *hfq*-deletion mutant during infection. Immunity against *B. abortus* involves antigen-specific T-cell activation, CD4+ and CD8+ T cells, and humoral responses, which mediate the acquired immunity against *B. abortus* infection in murine model (Fretin et al., 2005; Baldwin and Goenka, 2006; Rolán and Tsolis, 2008).

*IL-2* and *IFN*-γ, are associated with Th1 responses, regulatory cells, and the stimulation of cellular immunity, whereas *IL-4* and *IL-10* are generally associated with Th2 responses and the stimulation of protective humoral responses (Allen and Maizels, 1997; Glimcher and Murphy, 2000; Goldingm et al., 2001). The production of these cytokines have been frequently correlated with an symbol of early event in the defense mechanisms against intracellular pathogens and protection in other studies evaluating vaccine efficacy against intracellular bacteria (Goldingm et al., 2001). To investigate changes in the host immune response, the expression of cytokine genes, including *IL-2, IFN*-γ, *IL-4*, and *IL-10*, were analyzed by quantitative reverse transcription-polymerase chain reaction (qRT-PCR), and induction of *IL-2, IFN*-γ*, IL-4*, and *IL-10*, were tested by indirect ELISA.

## Materials and methods

### Bacterial strains

*B. melitensis* 16 M was routinely cultured in rich medium Tryptic Soy Broth (TSB) or Tryptic Soy Agar (TSA). The construction of the *hfq* deletion mutant 16MΔhfq and 16MΔhfq-C have been reported previously (Cui et al., 2013). When necessary, antibiotics were added to a final concentration of 50 μg/mL kanamycin and 50 μg/mL gentamicin.

### Mice and ethics statement

Female 6–8-week-old BALB/c mice were obtained from the Animal Center of Military Medical Sciences. All animals were handled in strict accordance with the Experimental Animal Regulation Ordinances defined by the China National Science and Technology Commission; the study was approved by the animal ethics committee of the Beijing Institute of Disease Control and Prevention. The animals were provided with humane care and healthful conditions during their stay in the facility. All individuals who handled the animals received instructions in experimental methods and in the care, maintenance, and handling of mice, and were under the committee's supervision.

### Macrophage infection and RNA extraction

Murine macrophage-like RAW264.7 cells were used to assess the survival capability of 16M, 16MΔhfq, and 16MΔhfq-C. Briefly, monolayers of macrophages (2 × 10^6^ cells/well) were cultured in a 6-well plate for 16 h at 37°C in an atmosphere of 5% CO_2_, and then infected with 16M, 16MΔhfq, and 16MΔhfq-C at a multiplicity of infection (MOI) of 100. Forty five min after addition to macrophage monolayers, the cells were washed twice with phosphate-buffered saline (PBS), and then incubated with 50 μ g/mL gentamicin for 60 min to kill extracellular bacteria. Then, the cultures were replaced with Dulbecco's modified Eagle's medium (DMEM) containing 25 μ g/mL gentamicin. At 0, 24, and 48 h post-infection, the supernatant was discarded, the cells were serially diluted and plated on TSA, and the CFUs were counted after 5 days of incubation at 37°C. One milli liter of TRIzol® was added to the cells for each well. Then, total RNA was extracted as recommended by the manufacturer. RNA was isolated from uninfected RAW264.7 cells as a negative control. The experiment was repeated three times, significant differences between the parent strain and PBS are indicated. The standard deviation (SD) is indicated by error bars.

### Mouse infection and RNA extraction

Female 6–8-week-old BALB/c mice (*n* = 25 per group, 5 per time point) were infected intraperitoneally with 200 μL of bacterial suspension containing approximately 2 × 10^7^ colony-forming units (CFUs) of each *Brucella* strain in sterile PBS or 200 μL of PBS as a negative control. At 7, 14, 28, and 45 days post-infection, mice were sacrificed by cervical dislocation, and the spleens were removed aseptically and homogenized with PBS containing 0.1% (v/v) Triton™ X-100. Half the homogenates were serially diluted and plated on TSA, and the CFUs were counted after 5 days of incubation at 37°C. Simultaneously, 1 mL of TRIzol® was added to the remaining homogenates for total RNA extraction.

### Immunization and virulence strain challenge

Female 6–8-week-old BALB/c mice (*n* = 20 per group, 5 per time point) were immunized intraperitoneally with a bacterial suspension containing approximately 2 × 10^7^ CFU of 16MΔhfq or PBS (negative control). Forty five days post the immunization, the mice were challenged with 1 × 10^5^ CFU of 16M. At 14 and 28 days post-challenge, the infected mice were sacrificed by cervical dislocation, and the spleens were removed aseptically and homogenized with 1 mL of PBS containing 0.1% (v/v) Triton™ X-100. Half the homogenates were serially diluted and plated on TSA, and the CFUs were counted after 5 days of incubation at 37°C. Total RNA was isolated from the remaining homogenates with TRIzol®.

### Quantitative RT-PCR

RNA samples were treated with DNase I (Promega) to remove contaminating genomic DNA. The RNA quantity and quality were analyzed by using an ND-1000 spectrophotometer (Nanodrop Technologies). cDNA was generated from total RNA by using a random hexamer primer and the SuperScript™ II reverse transcriptase kit (Invitrogen), according to the manufacturer's instructions. β-Actin or 16S rRNA, both of which are constantly transcribed in bacteria and cells, was chosen as an internal control. RT-PCR were performed in 20-μL volumes containing 10 μL of 2 × SYBR® Green I Master Mix (Takara Biochemicals), 100 nM each primer, and 1 μL of cDNA. The thermo cycling conditions were as follows: 15 min at 95°C for pre-incubation, followed by 40 cycles of amplification (95°C for 30 s, 60°C for 30 s, and 72°C for 30 s). The primers used for the qRT-PCR are listed in Table 1. All primer sets showed standard curves with R^2^-values of >0.980, 90–110% reaction efficiencies, and only one peak in the dissociation curves. Relative transcriptional level was determined by the 2^−ΔΔCt^ method, as described previously (Wang et al., 2009) using the following equation: relative fold change (treatment/control) = 2^−ΔΔCt^, where ΔCt (gene of interest) = Ct (gene of interest) − Ct (reference gene of the same sample) and ΔΔCt (gene of interest) = ΔCt (treatment) − ΔCt (control). The level of 16S rRNA or β-actin was used as a reference to normalize the expression data for the target genes.

**Table 1:** **Primers used in this study**.

**Primer name**	**Sequence (5′–3′)**
omp25-RT-R	CAGCACCGTTGGCAGCAT
omp25-RT-F	GGCATAACCGGGTTCAGG
omp31-RT-R	TCGTCGGTGGTGTTCAGG
omp31-RT-F	CGAGGTCGGTGTAGAGGTATT
vjbR-RT-R	CGAGGTGGAGGACGAAGA
vjbR-RT-F	ATAATGCCGAGGGAAAGC
dnaK-RT-R	TGAAATGGCAGCCGATAA
dnaK-RT-F	AAGCGAGGTCTTGAGGG
gntR-RT-R	AAAATGACCGAAGCATCTGG
gntR-RT-FT	GCGGGAAATGGGACGAA
htrA-RT-R	TTTGGCGACGATAATAAGGTG
htrA-RT-F	ATGGCGAGAAGATGGCG
16srRNA-RT-R	CACTGGACCATTACTGACGC
16srRNA-RT-F	ACTAAGGGCGAGGGTTGC
hfq-RT-R	CTTCCTCGCCTTCAAACATC
hfq-RT-F	GCAAAATCTACAAGACCTCTTTCT
IL-2-RT-R	TCCAGAACATGCCGCAGAG
IL-2-RT-F	CCTGAGCAGGATGGAGAATTACA
IL-4-RT-R	GAAGCCCTACAGACGAGCTCA
IL-4-RT-F	ACAGGAGAAGGGACGCCAT
IL-10-RT-R	ACCTGCTCCACTGCCTTGCT
IL-10-RT-F	GGTTGCCAAGCCTTATCGGA
IFN-γ-RT-R	TGGCTCTGCAGGATTTTCATG
IFN-γ-RT-F	TCAAGTGGCATAGATGTGGAAGAA
β-actin-RT-R	CAATAGTGATGACCTGGCCGT
β-actin-RT–F	AGAGGGAAATCGTGCGTGAC

### Cytokine secretion and antibody detection by ELISA

Serum samples were obtained from immunized mice at 7, 14, 28, and 45 days before challenge, 14 and 28 days post-challenge. Secretions of *IL-2, IFN*-γ*, IL-4*, and *IL-10* in the sera were determined by cytokine indirect ELISA (iELISA) kit essentially as recommended by manufacturer (4A, Biotech, China). Serial dilutions of standards were detected to generate standard curves, which was then used for sample concentration calculation. Antibodies against *Brucella* whole cell lysates in sera samples from immunized mice were determined to evaluate humoral immune response. Sera samples were collected at 7, 14, 28, and 45 days post the immunization, and then IgG antibodies were determined. Briefly, 96-well plates were coated with 100 μl 10^9^ cfu/ml heat-killed 16M whole-cell antigen. After overnight incubation at 4°C, plates were washed once with 100 μl PBST buffer (PBS containing 0.05% Tween-20) and blocked with 200 μl blocking buffer (10% heat-inactivated FBS in PBS, pH = 7.4) for 2 h at 37°C. Mice serum samples were diluted 1:100 in the dilution buffer and added to wells in triplicate and incubated for 2 h at 37°C. Following three washes with PBST to remove the unbound antibody, 100 ul rabbit anti-mouse IgG-horseradish peroxidase conjugate of 1:4000 was added to each well and incubated at 37°C for 30 min. After two washes with PBS, 100 ul per well of TMB substrate solution was added and incubated at 37°C in darkness for 15 min. The reaction was stopped by adding 50 ul of H_2_SO4 and the absorbance was measured at 450 nm(OD450). Antibody levels (IgG) were expressed as the arithmetic mean ± SD of the OD, ^*^P < 0.001.

### Statistical analysis

Bacterial survival under *in vitro* stress conditions was expressed as the mean percent survival compared to untreated controls ± the standard deviation (SD). Statistical analysis was performed with Student's unpaired *t*-test. Bacterial survival in mice was expressed as the mean log_10_ CFU ± SD. The differences between groups were analyzed by analysis of variance (ANOVA) followed by Tukey's honestly significant difference post-test, by comparing all the groups to one another. For the qRT-PCR experiments, significance was calculated by the Wilcoxon signed-rank test. In all cases, a *P*-value of less than 0.05 was considered significant.

## Results

### *hfq* is induced during macrophage and mouse infection

**Figure 1:**
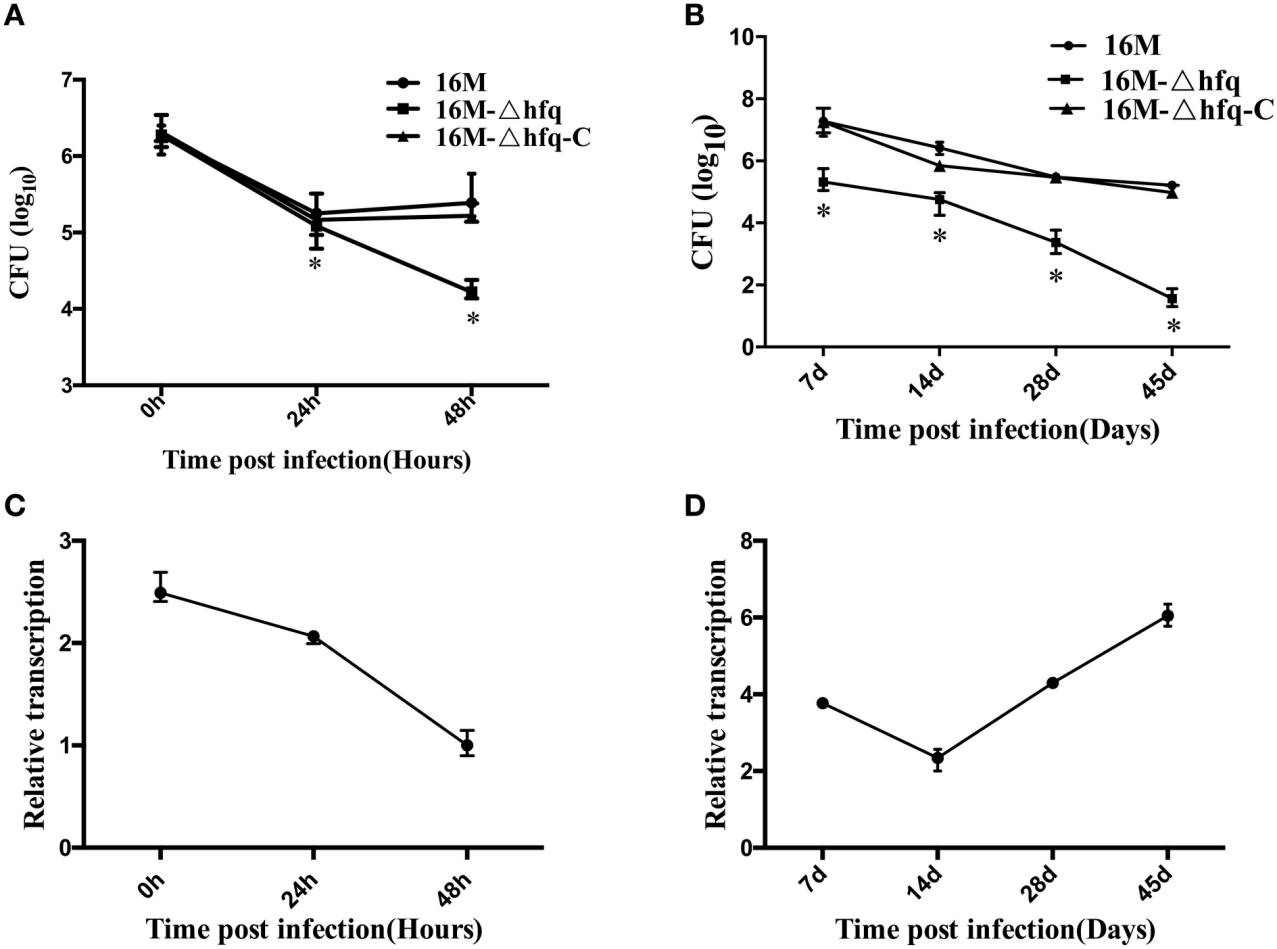
**Expression of *hfq* during macrophage and mouse infection**. RAW264.7 cells were infected with 16M, 16MΔhfq, and 16MΔhfq-C, at 0, 24, and 48 h post-infection, surviving bacteria number was determined **(A)**. BALB/c mice were infected with 16M, 16MΔhfq, and 16MΔhfq-C, at 7, 14, 28, and 45 days post-infection, surviving bacteria number was determined **(B)**. The relative transcription level of *hfq* during RAW264.7 **(C)** and BALB/c mice **(D)** infection was determined by RT-PCR. The SD is indicated by the error bar. Significant differences between the mutant and parent strain were indicated as follows: ^*^P < 0.001.

To examine the expression profile of *hfq* during infection, we first analyzed the transcription of *hfq* upon macrophage infection, because macrophages are one of the main target host cells for *Brucella*. Firstly, intracellular survival capabilities of the three strains were further confirmed. As shown in Figures 1A,B, survival of the *hfq* mutant was reduced in both macrophage and mouse model, being consistent with our previous results (Cui et al., 2013). Then, transcription of *hfq* during infection was analyzed. Macrophages were infected with 16M, and total RNA was isolated from the infected cells. Transcription of *hfq* during infection was determined by qRT-PCR. As shown in Figure 1C, compared to the expression level under *in vitro* conditions in TSB, *hfq* expression was enhanced by 2.5- and 2-fold at 0 and 24 h, respectively. However, at 48 h post-infection, *hfq* expression had decreased to the normal levels. This indicated that *hfq* is mainly induced during the early stage of infection. To further analyze the expression of *hfq* during infection, BALB/c mice were infected with 16M, and the transcription levels of *hfq* were determined at different time points. *hfq* induction was enhanced by approximately 4-fold at 7 days, but decreased to 2-fold at 14 days (Figure 1D). Then, at 28 and 45 days, *hfq* induction was enhanced by 4-and 6-fold, respectively. Together, these data indicated that *hfq* is induced during early infection in Raw264 cells, but in mice model, it is induced in the late stage of infection.

### *hfq* affects the expression of genes related to virulence during infection

**Figure 2:**
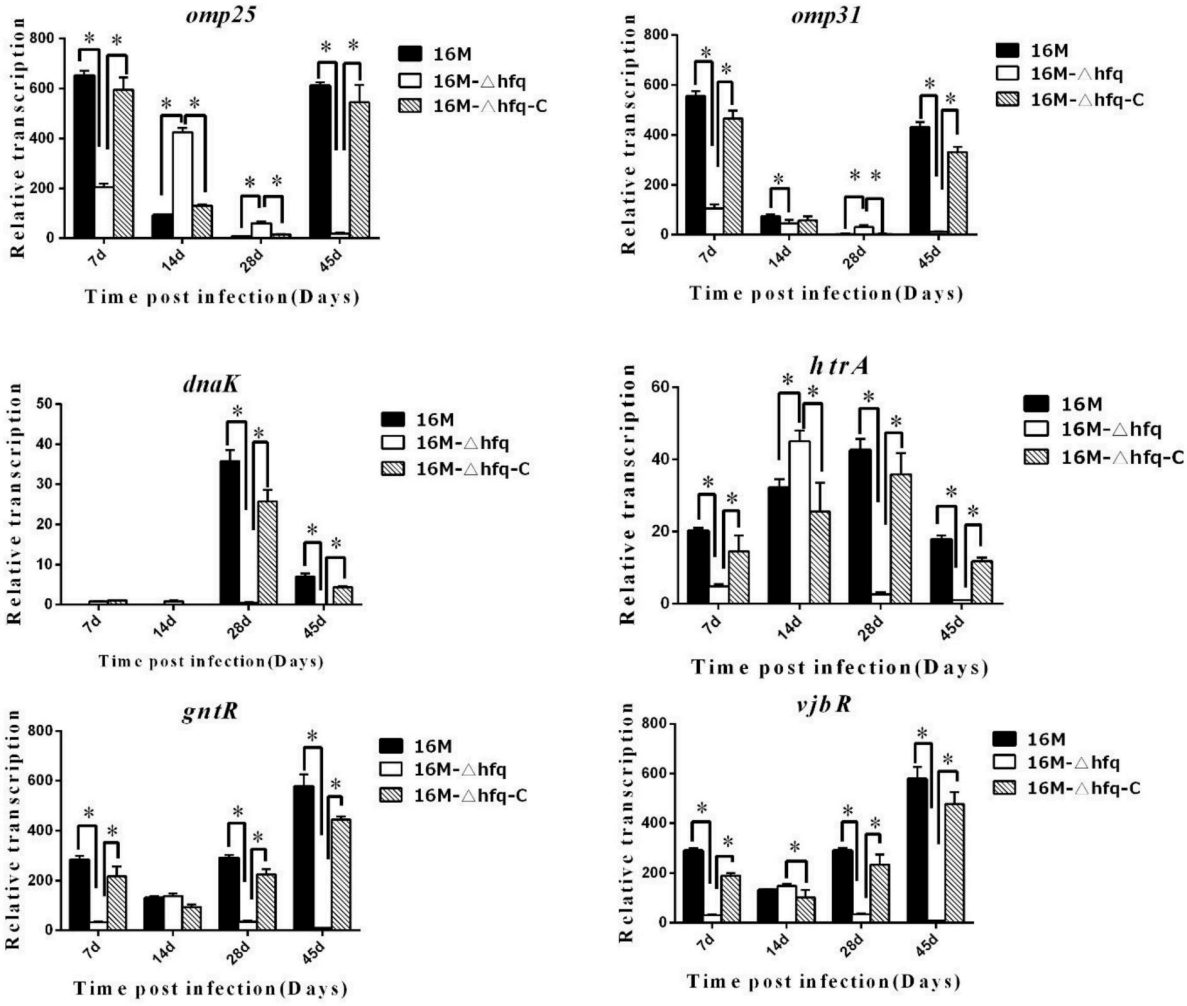
***hfq* regulates the expression of genes related to virulence during infection**. BALB/c mice were infected with *B. melitensis* 16M, 16MΔhfq, and 16MΔhfq-C, and then RNA was isolated from the spleens at 7, 14, 28, and 45 days post-infection. The relative transcription levels of virulence genes, *omp25, omp31, dnaK, htrA, gntR*, and *vjbR*, were determined by quantitative RT-PCR. The data were compared to the expression under standard *in vitro* conditions (TSB) of 16M. Significant differences between the mutant and parent strain were indicated as follows: ^*^P < 0.001.

The fact that *hfq* was induced during infection indicated that it plays important roles in intracellular survival, which is consistent with its involvement in *Brucella* virulence. As a small RNA chaperone, *hfq* regulates the expression of a large number of genes. Here, we analyzed the influence of *hfq* on genes related to virulence during mouse infection. Several important genes related to virulence, which are closely related to intracellular survival of *Brucella* (Elzer et al., 1996; Köhler et al., 1996; Edmonds et al., 2002; Haine et al., 2005; Caro-Hernández et al., 2007; Uzureau et al., 2007), including *omp25, omp31, dnaK, htrA, gntR*, and *vjbR*were selected, and their expression in 16M, 16MΔhfq, and 16MΔhfq-C during infection were analyzed. Interestingly, the expression levels of these genes differed with respect to the induction time points (Figure 2).

*omp25* is an outer membrane protein that is involved in the virulence of *Brucella*. In the wild-type strain 16M, expression of *omp25* was significantly induced at 7 and 45 days, while in the *hfq* mutant, *omp25* was induced at 7 and 14 days. *omp31* was also induced at 7 and 45 days in 16M, but not induced in the *hfq* mutant. *dnaK* was significantly induced in 16M at 28 days, but not in 16MΔhfq. *htrA* was induced in 16M at all 4 time points, but not at 7, 28, and 45 days in 16MΔhfq. Two other regulators, *gntR* and *vjbR*, were mostly induced at 45 days. For all these genes, their transcription of in 16MΔhfq-C was recovered when compared to that in 16MΔhfq. These data indicated that there is a significant disregulation of genes with known roles in virulence in the absence of *hfq*.

### Altered induction of cytokines by the *hfq* mutant during mouse infection

**Figure 3:**
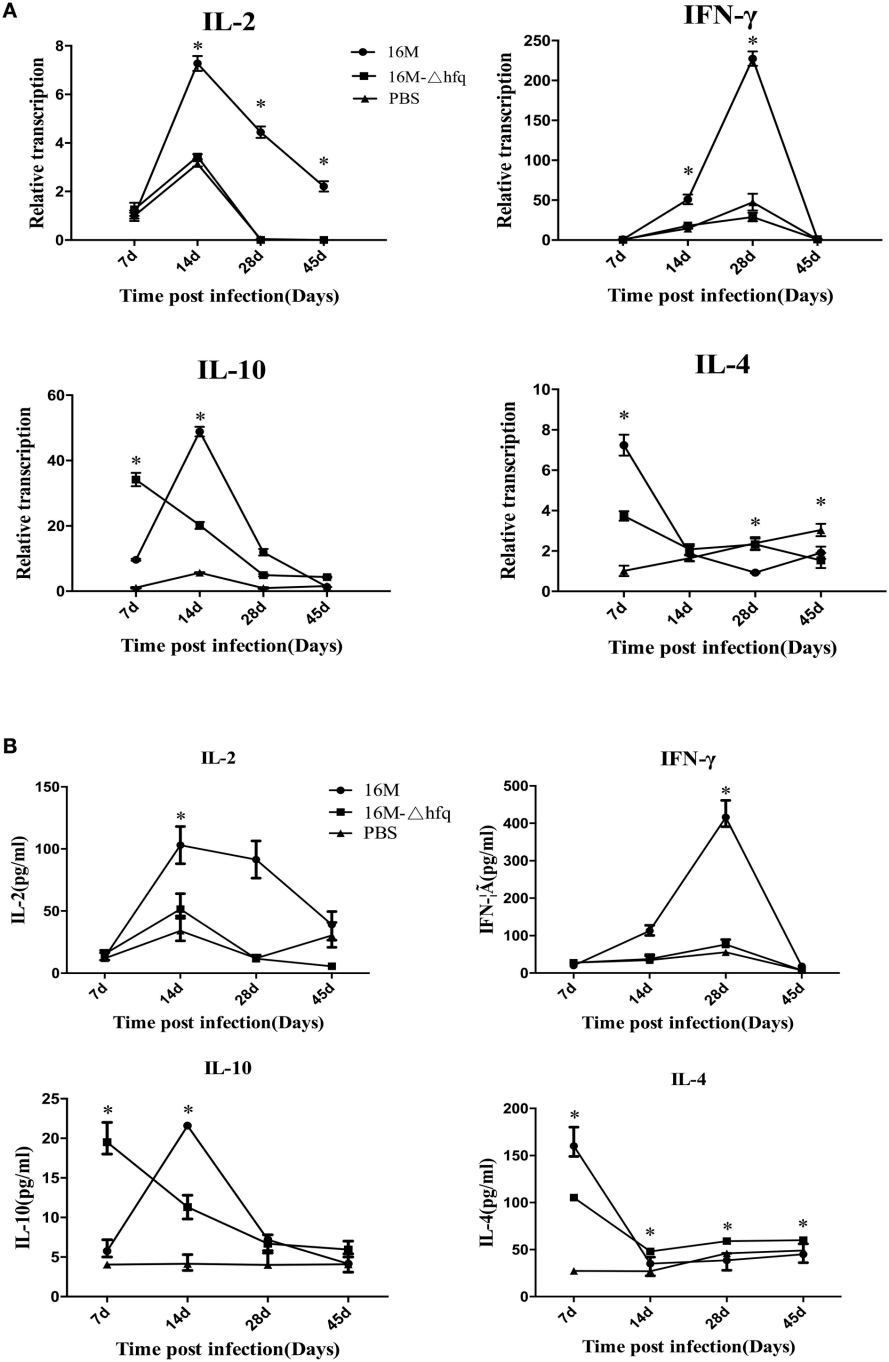
**Expression patterns of cytokine genes during *Brucella* infection**. BALB/c mice were infected with *B. melitensis* 16M, 16MΔhfq, or PBS as negative control, and then RNAs were isolated from the spleens at 7, 14, 28, and 45 days post-infection. The relative transcription levels of *IL-2, IFN*-γ, *IL-4*, and *IL-10*, were determined by quantitative RT-PCR **(A)**. Secretions of the four cytokines were detected by indirect ELISA **(B)**. The data were compared to the expression level of PBS at 7 days. Significant differences between the mutant and parent strain were indicated as follows: ^*^P < 0.001.

*Brucella* has the capability to inhibit the host immune responses that contribute to bacterial survival and replication in host. The above results indicated that disregulation of genes involved in virulence, in the absence of *hfq*, is a likely contributor to the significant attenuation observed for this strain *in vivo* (Roop et al., 2003; Caswell et al., 2012). Therefore, we hypothesized that the *hfq* deletion mutant might induce a different immune response in the host, particularly with respect to genes involved in protective immune response. To test this hypothesis, we analyzed the expression of *IL*-2 and *IFN*-γ, which are representative cytokines of Th1 immune response. As shown in Figure 3A, transcription analysis showed that the expression level of *IL*-2 increased from 7 to 14 days in the 16M group, while very low expression was detected in the *hfq*-mutant and PBS-control groups at 28 and 45 days. In contrast, the expression level of *IFN*-γ increased at 14 days and peaked at 28 days, and then decreased to a nearly undetectable level at 45 days in the 16M group. Compared to the levels in the wild-type group, the expression level of *IFN*-γ in the *hfq*-mutant was significantly reduced at 14 and 28 days. *IL-4* and *IL-10* are representative cytokines of the Th2 immune responses that inhibit Th1 response. Expression of *IL*-4 peaked at 7 days, and decreased with time in both the 16M and *hfq*-mutant groups. Expression of *IL-10* was significantly higher (by approximately 3-fold) in the *hfq*-mutant group than in the wild-type group at 7 days; however, the levels decreased at 14 and 28 days. Furthermore, secretion of cytokines was also detected by indirect ELISA. As shown in Figure 3B, secretion of *IL-2* in mice infected by *hfq*-mutant was lower than that by 16M at 14, 28, and 45 days. At 7 days, secretion level of *IL-4* and *IL-10* in *hfq* mutant group was lower than that in the 16M group. There was a good correlation between the transcription and secretion levels of the four cytokines.

### Infection with *hfq* mutant induces protective immune responses and antibodies

**Figure 4:**
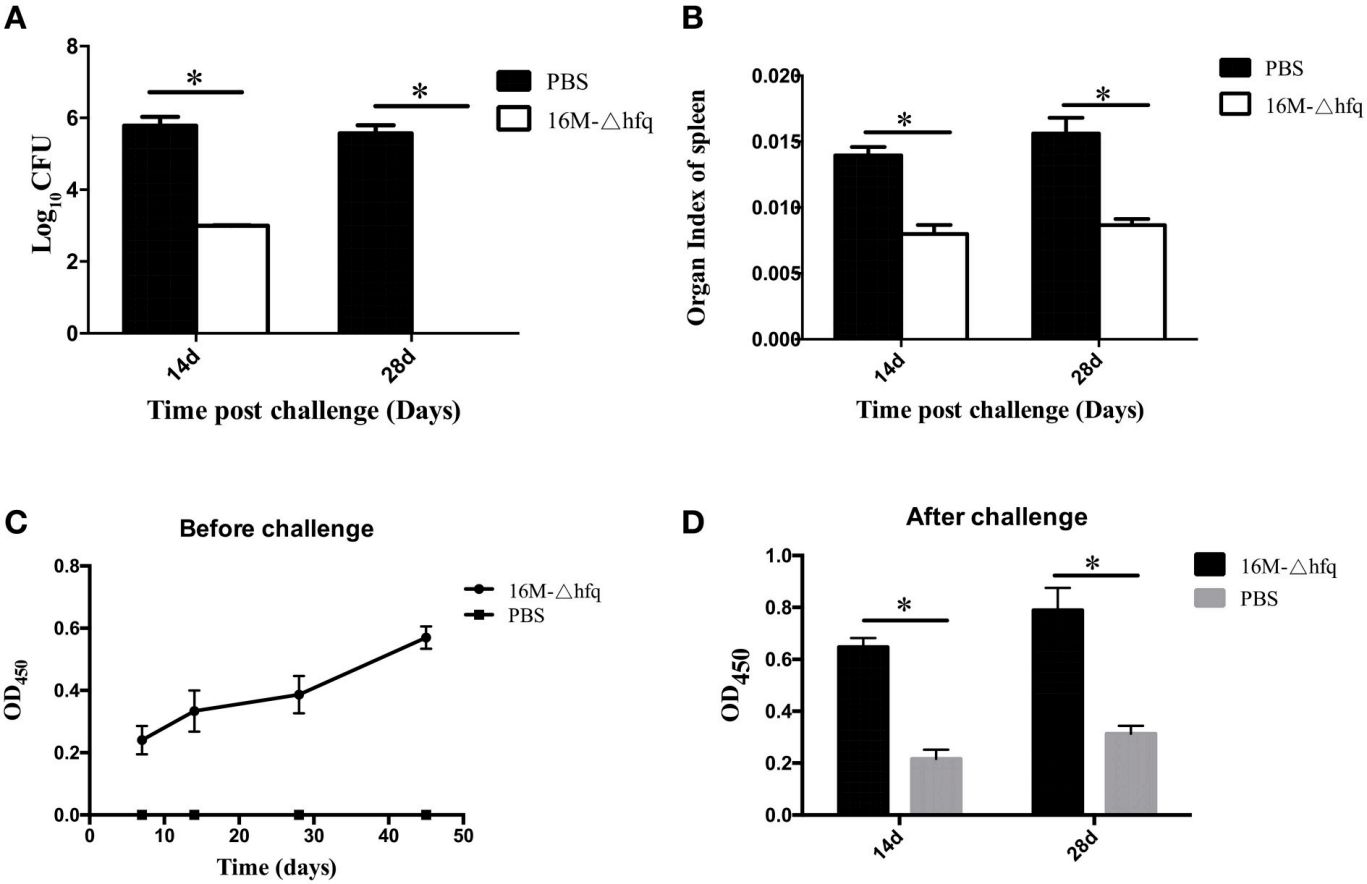
**Protection efficacy and antibody induction of the *hfq*-deletion mutant**. BALB/c mice were immunized with 16MΔhfq or PBS (negative control), and at 45 days post-immunization the mice were challenged with 16M. Fourteen and twenty eight days post the challenge, bacteria were isolated from the spleens and enumerated **(A)**, and organ indexes were calculated **(B)**. Antibody levels were detected at 7, 14, 28, and 45 days before challenge **(C)**, 14 and 28 days post-challenge **(D)**. The data were expressed as the mean log_10_ CFU ± SD (*n* = 5). Significant differences between the mutant and parent strain were indicated as follows: ^*^P < 0.001.

To test whether the *hfq* mutant induced protective immune responses, the mice were challenged with the virulent strain 16M. At 14 days, approximately 10^6^ CFU per mouse were isolated in the PBS group, while only 2.5 log CFU were isolated from *hfq*-immunized mouse. At 28 days, 5.8 log CFU were isolated from the PBS (control) group, but no bacteria were isolated for the *hfq*-mutant group (Figure 4A). This indicated that immunization with *hfq*-mutant induced protective immune response in the mice. Organ index refers to the weight of the spleen divided by the weight of the mouse (Figure 4B). Organ index analysis revealed that, compared to the PBS-treated mice, the *hfq*-mutant-immunized mice exhibited significantly lower organ indices both at 14 and 28 days post-infection. Antibody responses before and after challenge were also determined. As shown in Figure 4C, antibodies induced by 16MΔhfq increased slightly from 7 to 45 days. At 14 and 28 days post-challenges, antibody levels of the *hfq* mutant immunized mice were significantly higher that of PBS control (Figure 4D). This indicated that challenge of immunized mice with virulent strain induced significant *Brucella*-specific antibodies.

### Expression of cytokine genes by the mice after challenge

**Figure 5:**
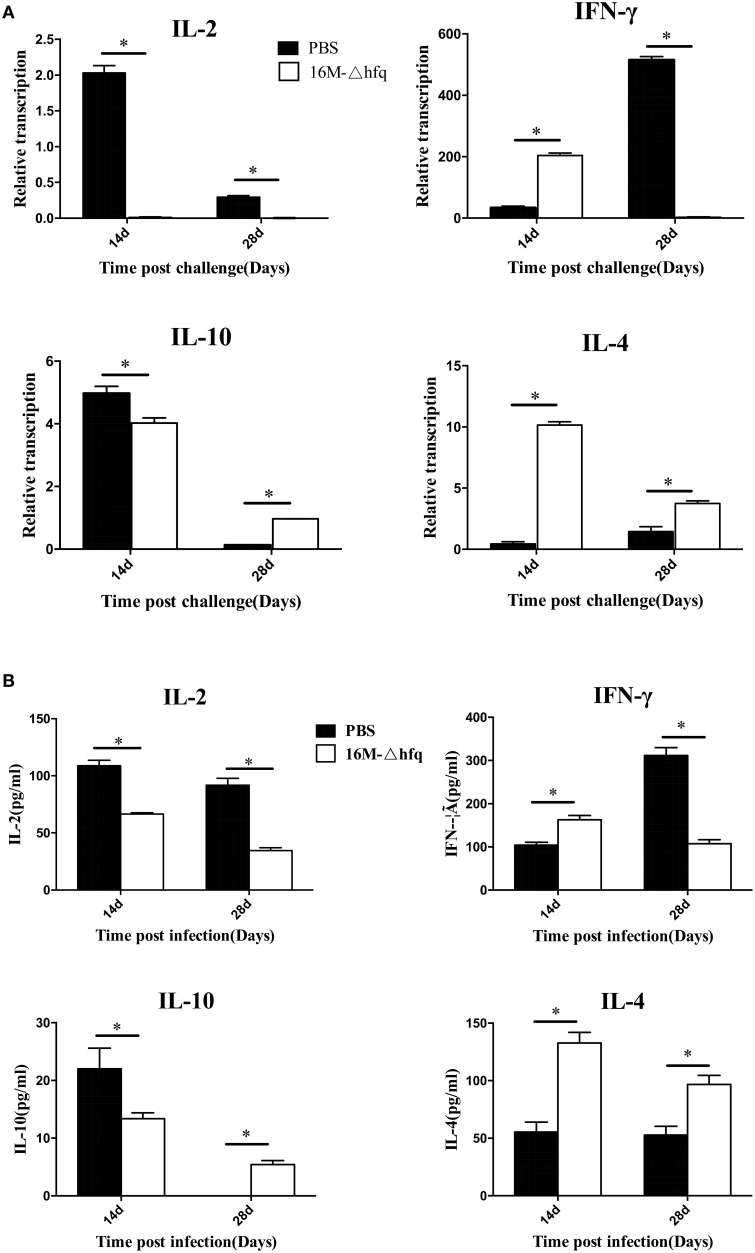
**Expression and secretion pattern of cytokine after the challenge**. Spleens were isolated from challenged mice at 14 and 28 days post-infection, the relative transcription levels **(A)** and secretion level **(B)** of *IL-2, IFN*-γ, *IL-4*, and *IL-10*, and were determined. The transcription levels were compared to each cytokine expression level of PBS group at 7 days. Significant differences between the mutant and parent strain were indicated as follows: ^*^P < 0.001.

The expression levels of cytokine genes were analyzed by qRT-PCR. At 14 days post-challenge, the expression of *IL*-2 and *IL*-10 was down regulated in mice of 16MΔhfq group, while expression of *IL-4* and *IFN*-γ was up regulated (Figure 5A). The expression level of *IL*-4 was higher in mice of the *hfq*-mutant group than that in the PBS group at 14 and 28 days. *IL*-10 was significantly induced at 14 days, but was down regulated at 28 days. *IFN*-γ was differentially induced between the two groups; the *hfq*-mutant group is about 18-fold that in the PBS group at 14 days, while the PBS group is 400-fold that of the *hfq*-mutant group at 28 days. Cytokine secretion analysis also showed levels of secreted cytokines also differed between PBS and *hfq*-mutant (Figure 5B). The secretion trends of the four cytokines were consistent with those of transcription levels between the two groups, but the extent differed.

## Discussion

*Brucella* is an intracellular bacterial pathogen. The intracellular survival capability of *Brucella* is one of its most important virulence mechanisms (Celli, 2006). To survive in host cells, *Brucella* has evolved complicated strategies to adapt to changing environments through the regulation of gene expression (Elzer et al., 1996; Köhler et al., 1996; Edmonds et al., 2002; Haine et al., 2005; Caro-Hernández et al., 2007; Uzureau et al., 2007; Wang et al., 2010). Noncoding small RNAs play important regulatory roles in the interactions between pathogens and hosts. Recent studies have revealed that noncoding small RNAs might play regulatory roles across species or even biological kingdoms (Kanneganti et al., 2006; Zhang et al., 2012). Hfq is a bacterial Sm-like protein that functions as a chaperone molecule and a post-transcriptional regulator of global gene expression. Hfq is essential for bacterial virulence and fitness (Kawamoto et al., 2006; Aiba, 2007; Sittka et al., 2008). In our previous studies, we demonstrated that *Brucella* encodes *hfq*, which in turn regulates gene expression related to virulence and intracellular survival in murine macrophages (Cui et al., 2013). *In vitro* transcriptome analysis revealed that *hfq* affects the expression of a large number of genes directly or indirectly (Saadeh et al., 2015). Comparative proteomics analysis led to the identification of differentially expressed proteins between wild-type and *hfq* deletion-mutant strains (Sittka et al., 2008; Cui et al., 2013).

In this study, we further investigated the expression of *hfq* and its effects on the expression of other genes related to virulence during infection. To examine the expression profiles of *hfq* during infection, we analyzed the transcription pattern of *hfq* upon murine macrophage infection and BALB/c mice infection. The results revealed that *hfq* expression was significantly induced during macrophage infection. *hfq* deletion mutant exhibits reduced survival and is attenuated in cultured macrophages. In addition, *hfq* was significantly induced at 0 h; however, the expression levels decreased at 24 and 48 h. This indicated that *hfq* plays important roles mainly at the early infection stage. These consequence is consistent with the results reported by Robertson GT and Martin RR (Bøggild et al., 2009) and Zhang et al (Zhang et al., 2013). In the case of infection in mice, the expression profiles of *hfq* followed a different pattern. *hfq* was upregulated by approximately 4-fold at 7 days, decreased to 2-fold at 14 days, and then increased at 28 and 45 days. In mice, an infection that lasts longer than 28 days is considered chronic infection. Therefore, it was inferred that *hfq* is induced at the both early and also late during chronic infection stages. Consistent with this hypothesis, the survival capability of the *hfq*-deletion mutant was reduced when compared with the wild-type strain. In particular, at the chronic infection stage, 16MΔhfq was nearly cleared from the infected mice, indicating that 16MΔhfq had lost its chronic infection capability (Zhang et al., 2013; Saadeh et al., 2015). Transcriptome analysis shows that Hfq act as either a direct or indirect contributor in gene expression under *in vitro* condition (Roop et al., 2003).

To further analyze the roles of *hfq* during infection, the expression patterns of several genes related to virulence were determined by qRT-PCR in 16M, 16MΔhfq, and 16MΔhfq-C during infection. Outer membrane proteins (OMPs) are essential for the maintenance of membrane integrity and selective permeability. Additionally, OMPs are often regulated by environmental signals, and they play important roles in bacterial pathogenesis by enhancing adaptability to various environments. OMPs of *Brucella* are involved in adaptation to environments both *in vitro* and *in vivo*. Two important OMPs, *omp25* and *omp31*, are involved in the virulence of *Brucella* (Jubier-Maurin et al., 2001; Caro-Hernández et al., 2007; Martín-Martín et al., 2008). In the present study, both *omp25* and *omp31* were found to be affected by *hfq* during infection, as evidenced by the fact that the expression levels of both genes were decreased in the *hfq*-deletion mutant. Expression of *omp25* and *omp31* in 16M was mostly induced at 7 and 45 days, and was higher than that in 16MΔhfq. This finding was consistent with the induction profile of *hfq* during mouse infection. This result indicated that *hfq* positively influence the expression of *omp25* and *omp31*, particularly at the early and chronic infection stages.

Global regulators are genes that regulate a large number of genes involved in various adaptations. A number of global regulators have been identified in *Brucella*, many of which are involved in *Brucella* virulence regulation (Elzer et al., 1996; Köhler et al., 1996; Edmonds et al., 2002; Haine et al., 2005; Caro-Hernández et al., 2007; Uzureau et al., 2007; Martín-Martín et al., 2008). On the basis of our previous transcriptome analysis, in this study, we analyzed the roles of *hfq* on six regulators related to virulence, including *omp25, omp31, dnaK* (Köhler et al., 1996), *htrA* (Elzer et al., 1996; Pallen and Wren, 1997), *gntR* (Haine et al., 2005), and *vjbR* (Uzureau et al., 2007; Arocena et al., 2012). Interestingly, the expression profiles of these genes differed significantly in terms of their induction time points and extents. Peak transcript levels were observed for *dnaK* at 28 days, *htrA* was mostly induced at 14 and 28 days, and *gntR* and *vjbR* were mostly induced at 45 days. Compared to the levels under standard *in vitro* conditions (TSB), the induction levels of these genes were increased up to hundreds of fold, implying that these genes are highly induced during mouse infection at specific infection stages. *dnaK* is a heat shock protein that regulates the expression of various factors in response to heat shock-related stimuli (Köhler et al., 1996, 2002). *htrA* is a regulator that is involved in oxidative stress responses (Pallen and Wren, 1997). *gntR* is a regulator usually related to metabolism (Fujita et al., 2007). *vjbR* is a regulator that functions in response to cell density changes (Uzureau et al., 2007). All four virulence genes sense and respond to environmental changes by regulating the expression of other genes. The different induction profiles of the four regulators implied that *Brucella* encounters varied environmental conditions during mouse infection. Compared to the induction levels in 16M, these genes were mainly down regulated in 16MΔhfq. These results indicated that the reduced survival capability 16MΔhfq is a consequence of the decreased induction levels of virulence regulators. Although these virulence related genes are altered in their expression level in the *hfq* mutant, it remains to be defined whether directly or indirectly by *hfq*-mediated regulation or if instead these genes are downregulated as a consequence of poor *in vivo* fitness.

To survive in host cells, *Brucella* has evolved the capability to inhibit host immune responses that are harmful to its survival. Deletion of *hfq* resulted in reduced survival and expression of genes related to virulence, implying that the deletion mutant might induce different immune responses. Th1 cells are involved in host defense against intracellular pathogens by producing *IFN*-γ, *TNF*-α, and *IL-2*, while Th2 cells are responsible for coordinating humoral immunity, are involved in host defense against extracellular parasites by secreting *IL-4, IL-5, IL-10*, and *IL-13* (Allen and Maizels, 1997; Glimcher and Murphy, 2000; Goldingm et al., 2001). In this study, we analyzed the humoral immune response and the cell-mediated immune response induced by infected mice and challenged mice. To test this hypothesis, the expression levels of four cytokine genes, including *IL*-2, *IFN*-γ, *IL*-4, and *IL*-10, were analyzed by qRT-PCR. Of the four cytokines, *IL-2* and *IFN*-γ mediate Th1-type immune response, while *IL-4* and *IL* -10 mediate Th2-type response. Both *IL*-2 and *IFN*-γ were maximally upregulated at 14 and 28 days in the 16M group. However, the induction levels of these genes were reduced in 16MΔhfq- and PBS-infected mice. *IL*-4 was upregulated by 7-fold at 7 days, and then downregulated at 14–45 days. *IL*-10 was upregulated by 10-fold at 7 days; the expression level peaked at 14 days, and then decreased at 28 and 45 days. Together, these results suggest that *IL*-4 and *IL*-10 were induced mostly at 7 and 14 days. Interestingly, the induction level of *IL*-10 in 16MΔhfq at 7 days was higher than that in 16M. To confirm the results, secretions of the four cytokines were also tested. The results showed that the secretion trends were consistent with the transcription changes, confirming the alteration of cytokine response induction. This finding also implied that, at this time point, 16MΔhfq induced a fierce Th2-type response. Early differences in cytokine responses contribute to a stronger Th1 polarization of the immune response in mice infected with wild-type *B. abortus* than in mice infected with the *hfq* mutant Although it is difficult to compare these results with those from previously published studies for differences in methodology, the trend is in agreement with previously relevant reports (Baldwin and Goenka, 2006; Kahl-McDonagh and Ficht, 2006; Rolán and Tsolis, 2008; Zhang et al., 2013).

The altered expression of virulence and cytokine genes implied that 16MΔhfq induced immune responses in mice different from that by 16M. To test whether this immune response protects against future infection, the infected mice were challenged with 16M. Surprisingly, compared with PBS-immunized mice, the bacterial number in the spleen of 16MΔhfq-infected mice was reduced by 2.5 logs at 14 days and nearly cleared at 28 days, which induced a great decline than before challenge. However, 16M group was approximately 6 logs at 14 and 28 days, the CFUs of spleen didn't much change than before. This indicated that infection with 16MΔhfq induced protective immunize responses against the virulent strain. These data are consistent with the results reported by Zhang et al. (2013). To further characterize the protection mechanisms, the expression patterns and production of various cytokines were analyzed. In the PBS group, *IL*-2 was upregulated by 2-fold at 14 days, while *IFN*-γ was markedly upregulated at 28 days. In contrast, *IL*-2 was nearly undetectable in the 16MΔhfq group, and *IFN*-γ was induced at 14 days in the 16MΔhfq group. *IL*-4 was found to be upregulated in the 16MΔhfq group, compared to the levels detected in the PBS group. In contrast to the study by Zhang and his colleagues, we detected cytokines at more time points; this might provide more comprehensive information on cytokine expression during infection. Besides, the production of *IFN*-γ and *IL-4* induced by 16MΔhfq group after challenge will give us more new ideas to explore the immune mechanism. Together, our data indicate that immunization with 16MΔhfq induced different cytokine profiles, which in turn conferred protection against challenge with the virulent strain.

In summary, in this study, we analyzed the expression profiles of *hfq* and other genes related to virulence during mice infection. The expression of virulence related genes are reduced in 16MΔhfq. Moreover, infection with 16MΔhfq induced different immune responses and conferred protection against future challenge by the virulent strain, indicating that 16MΔhfq might represent a good live attenuated vaccine candidate for brucellosis and further evaluation will be tested in other livestock.

## Author contributions

SL and ZC wrote the main manuscript text. ZC, ZZ, and GP helped conceive the project and designed the experiments. SL, CA, XX, JY, HR, MY, and YW executed the experiments, the other authors helped to revising it for important intellectual content. GP, ZC, and YW has decided the final approval of the version to be published. All authors have contributed to this review from their complementing areas of expertise. All authors read and approved the final manuscript.

### Conflict of interest statement

The authors declare that the research was conducted in the absence of any commercial or financial relationships that could be construed as a potential conflict of interest.
